# MXene-GaN van der Waals metal-semiconductor junctions for high performance multiple quantum well photodetectors

**DOI:** 10.1038/s41377-021-00619-1

**Published:** 2021-09-02

**Authors:** Lingzhi Luo, Yixuan Huang, Keming Cheng, Abdullah Alhassan, Mahdi Alqahtani, Libin Tang, Zhiming Wang, Jiang Wu

**Affiliations:** 1 Institute of Fundamental and Frontier Sciences, University of Electronic Science and Technology of China, 610054 Chengdu, China; 2 Materials Department, University of California, Santa Barbara, California 93106 USA; 3 King Abdulaziz City for Science and Technology (KACST), Riyadh, Saudi Arabia; 4 Kunming Institute of Physics, 650223 Kunming, Yunnan China; 5 State Key Laboratory of Electronic Thin Films and Integrated Devices, University of Electronic Science and Technology of China, 610054 Chengdu, Sichuan China

**Keywords:** Optoelectronic devices and components, Applied optics

## Abstract

A MXene-GaN-MXene based multiple quantum well photodetector was prepared on patterned sapphire substrate by facile drop casting. The use of MXene electrodes improves the responsivity and reduces dark current, compared with traditional Metal-Semiconductor-Metal (MSM) photodetectors using Cr/Au electrodes. Dark current of the device using MXene-GaN van der Waals junctions is reduced by three orders of magnitude and its noise spectral intensity shows distinct improvement compared with the traditional Cr/Au–GaN–Cr/Au MSM photodetector. The improved device performance is attributed to low-defect MXene-GaN van der Waals interfaces. Thanks to the high quality MXene-GaN interfaces, it is possible to verify that the patterned substrate can locally improve both light extraction and photocurrent collection. The measured responsivity and specific detectivity reach as high as 64.6 A/W and 1.93 × 10^12^ Jones, respectively, making it a potential candidate for underwater optical detection and communication. The simple fabrication of MXene-GaN-MXene photodetectors spearheaded the way to high performance photodetection by combining the advantages of emerging 2D MXene materials with the conventional III-V materials.

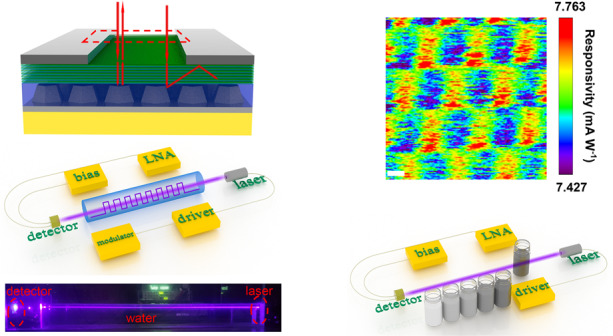

## Abstract (web summary)

A MXene-GaN van der Waals Metal-Semiconductor Junctions based High Performance Multiple Quantum Well Photodetector was demonstrated, which shows great potential for underwater wireless optical communication and turbidity sensing.

## Introduction

The arrival of the era of Internet of Things (IoT) has sparked intense interest of photodetectors (PD) as they are widely used in sensing^1^, detection^2^, data transport, and processing^3^. To meet the demands of these applications, high sensitivity, high bandwidth, less thermal loss, and less electromagnetic interference are required^4^. In terms of typical device structures, a photodetector can be categorized as follows: Schottky barrier detectors, metal-semiconductor-metal detectors, p-i-n (PIN) photodiodes, avalanche photodiode (APD), photoconductors, and phototransistors^5,6^. MSM photodetectors, which consist of two back to back Schottky contacts, have received much attention for their high response speed^7^. In addition, it is easy to fabricate and integrate with field effect transistor (FET) technology as they can share the same Schottky contacts of FET gates^8^. However, MSM photodetectors often exhibit high dark currents. Typically, a metal or metal alloy is employed to form the Schottky contact with semiconductors. The deposition of metals induces chemical disorder and defect states at the metal-semiconductor interfaces, leading to significant reverse tunneling currents^9^. A number of methods have been proposed to solve this issue, such as utilizing a wide bandgap semiconductor to increase the rectifying Schottky barrier heights^10^, repelling the injected electrons from the metal through confined carrier gases^11^, or reducing density of states in the semiconductor. However, MSM photodetectors based on Schottky junctions show inferior signal-to-noise ratios compared to PIN diodes. Additionally, opaque metals are usually placed on top of the active light absorption region, which will reflect part of the incident light thus reduce the responsivity. Transparent electrodes, such as ITO, TiN, RuO, and IrO are limited by their high cost and brittleness. Backside illumination was proposed to solve this problem but will increase the complexity in device processing and chip packaging^12^.

MXene, a new type of two-dimensional (2D) materials discovered in 2011^13^, is promising to relieve what traditional MSM photodetectors have suffered. MXenes are 2D transition metal carbides or carbonitrides, with a general formula *M*_*n* + 1_*X*_*n*_*T*_*x*_ (*n* = 1, 2, 3), where *M* is an early transition metal, *X* is a C and/or N atom, and *T*_*x*_ are the surface terminating functional groups, mostly –O, –OH, and/or –F^14^. This unique 2D materials have many charming properties, such as metallic conductivity^15^, mechanical flexibility^16^, hydrophilia^17^, good transmittance^18^, and chemical stability^19^, enabling MXenes to be processed in solution at low temperatures and under ambient conditions. Additionally, the different surface terminating functional groups will affect the electrostatic potential and electronic structures of MXenes^20^, thus shifting its work function from 1.6–6.2 eV^21^. The widely tunable work function makes MXene a great candidate for ohmic or Schottky contacts with various semiconductor materials. More importantly, 2D materials consist of in-plane covalently bonded atomic layers that interact weakly with each other in the out-of-plane direction. When deposited on bulk semiconductor materials, MXene-semiconductor van der Waals junctions formed at the interface are free of chemical disorder and defect states, which could avoid the Fermi level pinning effect and reduce the reverse tunneling currents^9^. To date, MXenes have been widely researched in fields of photodetectors^2^, sensors^22,23^, electromagnetic interference shielding and absorption^24^, photoelectrochemical catalysis^25^, optical transmission, and modulation^26,27^.

In this paper, an InGaN/GaN multiple quantum well (MQW) photodetector using the MXene-GaN-MXene structure was proposed. To avoid a large density of threading dislocations (TDs) and basal stacking faults (BSFs) between GaN epilayers and the sapphire substrate, a patterned sapphire substrate (PSS) was employed as the growth substrate. The growth of GaN on PPS can promote the epitaxial lateral overgrowth (ELOG) mode and consequently reduce the defect density in GaN epilayers^28,29^. A facile drop casting method using a shadow mask was adopted to fabricate the MXene-GaN-MXene structure. The responsivity and specific detectivity reach as high as 64.6 A/W and 1.9 × 10^12^ Jones, respectively under *λ* = 405 nm illumination, exceeding the performance of its Cr/Au counterpart and making it suitable for underwater detection and communication. Moreover, the GaN materials grown on the patterned sapphire substrate offer interesting localized photon extraction and photocurrent collection, which may be useful for developing a new type of focal plane arrays. At last, a simple underwater wireless optical communication system (UWOCS) and a turbidity sensing system (TSS) are demonstrated to exhibit its potential in underwater communication and detection.

## Results

**Fig. 1:**
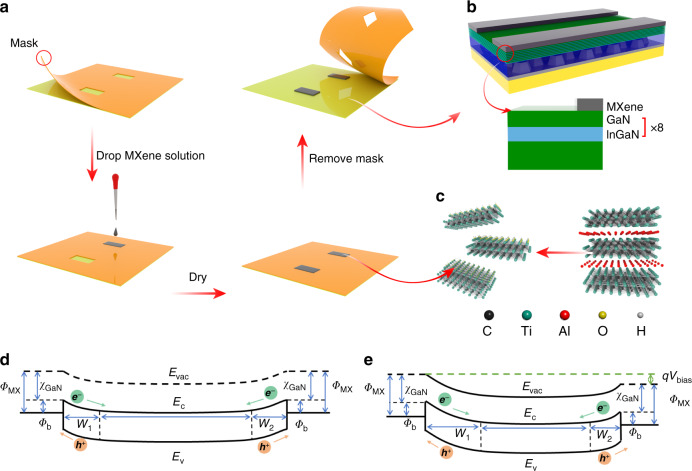
Fabrication process, device structure, and energy band diagram. **a** Process flow diagram of the facile drop casting method. **b** Schematic diagram of the proposed PD prepared on the patterned sapphire substrate. **c** Schematic illustration of the synthesis and structure of Ti_3_C_2_T_x_ MXene. Sketch of the energy band diagram of devices under **d** zero bias and **e** moderate bias.

Thanks to the hydrophilia and chemical stability of Ti_3_C_2_T_*x*_ MXene, the low-cost facile drop casting method can be adopted to fabricate the MXene-GaN-MXene structure as illustrated in Fig. 1a, b. Initially, an aqueous solution of single-layer Ti_3_C_2_T_x_ MXene nanosheets was synthesized by chemically etching the Al layers of the Ti_3_AlC_2_ MAX phase. The exfoliated titanium carbide layers consist of unit cells with three titanium atoms bonding to two carbon atoms, as illustrated in Fig. 1c. A polyvinyl chloride (PVC) electrostatic film was employed as the shadow mask, on which the electrode patterns were carved. Through the shadow mask, the as-prepared Ti_3_C_2_T_x_ MXene solution was dropcasted on the surface of an InGaN/GaN MQW sample. The PVC electrostatic film was sequentially removed after the formation of Ti_3_C_2_T_x_ MXene electrodes. At the same time, a Cr/Au–GaN-Cr/Au MSM photodetector was fabricated as a reference by the conventional device process using the same mask. As shown in Fig. 1d, e, when a moderate voltage applying to the detector, the Schottky junction at the cathode side is reverse biased. Therefore, the depletion width at the cathodes side ($W_1$) is lager than at the anode side ($W_2$) and most of the electric potential drops at the cathode side. In this case, the dark current of the MSM PD is dominated by the properties of the reverse biased cathode described by the Richardson and Dushman equation:

1 $$I_{{{{\mathrm{dark}}}}} = A^ \ast \times T^2 \times Ae^{\frac{{ - q{\Phi}_{{{\mathrm{b}}}}}}{{k_{{{\mathrm{B}}}} \times T}}} \times e^{\frac{{q{\Delta}{\Phi}_{{{{\mathrm{im}}}}}}}{{k_{{{\mathrm{B}}}} \times T}}}[{{{\mathrm{Amp}}}}]$$

where $A$ is the area of the photodetector, $A^ \ast$ is the Richardson constant, ${\Phi}_{{{\mathrm{b}}}}$ is the Schottky barrier height, ${\Delta}{\Phi}_{{{{\mathrm{im}}}}}$ is the barrier lowering due to the image force, and other symbols have their usual meaning. According to Eq. (1), ${\Phi}_{{{\mathrm{b}}}}$ is a significant parameter to determine the dark current. In the ideal case that the semiconductor has no surface states, ${\Phi}_{{{\mathrm{b}}}}$ is the difference of the metal work function (${\Phi}_{{{\mathrm{m}}}}$) and semiconductor electron affinity ($\chi$). However, due to the large number of surface states caused in the conventional deposition process of metals, the Fermi level is pinned at the interface, resulting in barrier heights that are relatively independent of ${\Phi}_{{{\mathrm{m}}}}$. Specifically, for the Cr/Au–GaN interface, the conventional device processing tends to cause localized deep defect donors related to N vacancy or its complex. Deep defect donors near the interface narrow the Schottky barrier width and thus increase the dark current through thermionic field emission.^30^ The leakage current mechanism model in GaN–Au/Cr Schottky interfaces accounting for the large dark current is given in the Part I of the supplementary information. These disadvantages of conventional metal-semiconductor interfaces can be relieved by using the van der Waals junctions since there are fewer defect states at the interface as mentioned afore, which could avoid deep donors, the Fermi level pinning effect, and hence reduce the reverse tunneling currents. Therefore, the dark current of the MXene–GaN–MXene MQW PD is expected to be much lower than that of the Cr/Au–GaN–Cr/Au MQW PD.

**Fig. 2:**
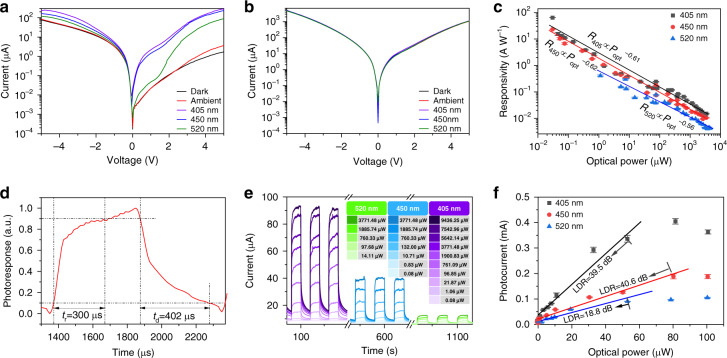
Photoresponse characteristics. *I*–*V* curves in the logarithmic coordinate axis of **a** the proposed PD and **b** the Au/Cr-GaN-Au/Cr MQW PD measured in the dark, ambient, and under illumination with different wavelengths (405 nm, 450 nm, and 520 nm). **c** Responsivity of the proposed PD versus the incident optical power in the logarithmic coordinate. The fitted line and parameters are marked in the figure. **d** Normalized transient photocurrent response of the proposed PD. **e** Time-dependent photoresponse under various optical power in different wavelengths (405 nm, 450 nm, and 520 nm) of the proposed PD at a +5 V applied bias. **f** Linear dynamic ranges of the proposed device under illumination with different wavelengths (405 nm, 450 nm, and 520 nm).

Based on these considerations, systematic characterizations were carried out on the MSM devices. Figure 2a, b show the current–voltage (*I*–*V*) characteristics of the proposed PD and the Cr/Au–GaN–Cr/Au MQW PD in the logarithmic coordinate axis measured in the dark, ambient, and under *λ* = 405 nm, 450 nm, and 520 nm illumination, respectively. Figure S1a, b exhibit typical *I*–*V* curves of MSM photodetectors, implying rectifying Schottky barrier exists at both the MXene–GaN and Cr/Au–GaN interfaces. As expected, the dark current of the Cr/Au–GaN–Cr/Au MQW PD has exceeded 1 mA under +5 V bias and the photocurrent is almost overwhelmed by the dark current as shown in Fig. 2b. Four more Cr/Au–GaN–Cr/Au MQW PDs were fabricated and without exception, all exhibit higher dark currents as shown in Fig. S1c–f. This is because the MQWs are placed in close proximity to the surface with a GaN cap layer of only 10 nm. The surface defects introduced during the conventional evaporation of Cr/Au layers assist charge carriers to tunnel through the GaN cap layer and lead to a significantly increased dark current. Thanks to the high quality MXene-GaN van der Waals junctions that are free of chemical disorder and less defect states, dark current of the MXene-GaN-MXene PD is reduced down by three orders of magnitude under +5 V bias, and a distinct photocurrent can be observed.

The photoresponsivity ($R$) versus incident optical power ($P_{{{{\mathrm{opt}}}}}$) at different wavelengths are shown in Fig. 2c. The responsivity was measured as high as 64.62 A/W under an incident optical power of 0.3 µW at the wavelength of 405 nm. However, the responsivity drops to 0.07 A/W, when the incident optical power was increased to 3771.48 µW. The illumination with light sources of 450 nm and 520 nm exhibited similar behaviors. The responsivity as a function of optical power can be fitted well with the power function and the fitted power exponents are −0.61, −0.62, and −0.56, respectively, as shown in Fig. 2c. The responsivity (*R*) of a photodetector measuring the electrical output for per unit optical input can be expressed as:

2 $$R = \frac{{I_{{{{\mathrm{ph}}}}}}}{{P_{{{{\mathrm{opt}}}}}}} = \frac{{\lambda q}}{{hc}} \times \eta \times G[AW^{ - 1}]$$

where $I_{{{{\mathrm{ph}}}}}$ is the photocurrent, $\eta$ the quantum efficiency, $\lambda$ the excitation wavelength in vacuum, $q$ the unit charge, $G$ the photoconductive gain, and other symbols have their usual meanings. Assuming $\eta$ equals 100%, the highest $G$ is estimated as 198. $G$ equals $(\tau _{{{\mathrm{p}}}}/\tau _{{{\mathrm{t}}}}) \cdot (1 + \mu _{{{\mathrm{n}}}}/\mu _{{{\mathrm{p}}}})$, where $\tau _{{{\mathrm{p}}}}$, $\tau _{{{\mathrm{t}}}}$, $\mu _{{{\mathrm{p}}}}$, $\mu _{{{\mathrm{n}}}}$ are the recombination lifetime of holes, transmit time, hole mobility, and electron mobility, respectively. The high value of $G$ is attributed to minority carrier traps, i.e., the hole traps, in the nitride materials when excited by weak light. Hole traps refer to the defect levels where the hole capture probability is much higher than the electron capture probability. When a hole fall into a hole trap, it cannot recombine with an electron directly. It must be excited into the valence band and then recombine with an electron, which is a relatively long relaxation process and results in unbalanced carrier concentration.^31^ This process is equivalent to increasing $\tau _{{{\mathrm{p}}}}$, giving rise to a high $G$. With gradually increasing the optical power, the hole traps are eventually saturated by the photoexcited holes and the rest of excess holes experience a short $\tau _{{{\mathrm{p}}}}$. Therefore, the average lifetime $\tau _{{{\mathrm{p}}}}$ decreases, leading to a lower responsivity when detecting strong light. As shown in Fig. S2a, the curve of ${\Delta}I_{{{{\mathrm{ph}}}}}/{\Delta}P_{{{{\mathrm{opt}}}}}$ versus $P_{{{{\mathrm{opt}}}}}$ shows distinct rise when detecting light at low optical power, which is a strong evidence for the carrier dynamics stated above. As for the Au/Cr counterpart, the deep defect donor levels often act as recombination center, suppressing the minority trap assisted processes for a high $G$, as shown in Fig. S2b.

Dynamic characteristics of the proposed PD was investigated by continuously modulating the incident light. Figure 2d shows the photocurrent rising and decay curves at 5V bias. The rise time ($t_{{{\mathrm{r}}}}$) and decay time ($t_{{{\mathrm{d}}}}$) are defined as the time span that photocurrent rises from 10% to 90% and decays from 90% to 10%, respectively. Though the PD sample has a large device area (~9.6 mm^2^) and long channel (~0.54 mm), it exhibits short $t_{{{\mathrm{r}}}}$ of 300 µs and $t_{{{\mathrm{d}}}}$ of 402 µs. The rising and decay curve contain a fast component that current rises from 0 to 70% and decay from 100% to 50% and a slow component that continue to rise from 70% to 100% and decay from 50% to 0. The two components are attribute to the short carrier lifetime outside traps and long carrier lifetime in traps. Since most photodetectors are lowpass system, the 3 dB bandwidth can be estimated by the $t_{{{\mathrm{r}}}}$:

3 $$f_{3{{{\mathrm{dB}}}}} \cong \frac{{0.35}}{{t_{{{\mathrm{r}}}}}}[Hz]$$

The $f_{3{{{\mathrm{dB}}}}}$ of our photodetector is estimated as 1167 Hz. The time-dependent photoresponse to incident light of different wavelengths in different optical power is depicted in Fig. 2e. The lowest possible optical detection power is as low as 80 nW for 405 nm and 450 nm light sources but is 14.11 µW for the 520 nm light source due to a lower absorption efficiency of InGaN/GaN QWs. Nonetheless, the high responsivity confirms good interface quality of the MXene-GaN van der Waals junctions. At the same time, the device exhibits a relatively repeatable and stable photocurrent under all measured optical power. As shown in Fig. 2f, the proposed PD has a large linear dynamic range of 40.6 dB, implying the dynamic range spans more than four orders, which is comparable to the GaN based photodetector reported in literature^32^.

**Fig. 3:**
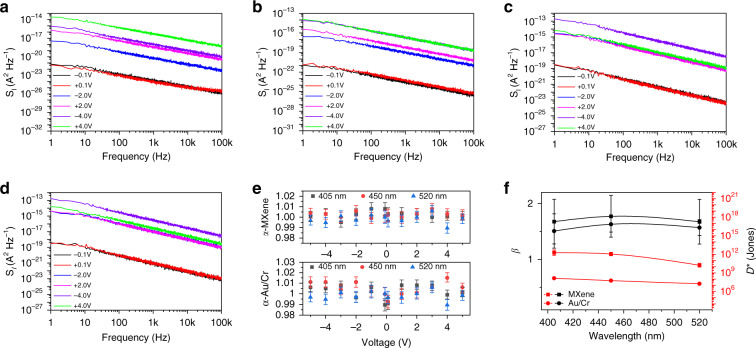
Noise characterization. Noise spectral density of the proposed PD **a** in the ambient **b** under illumination of a 405-nm lasing diodes. Noise spectral density of the Cr/Au–GaN–Cr/Au MQW PD **c** in the ambient and **d** under illumination of a 405-nm lasing diodes. **e** The fitting parameters *α* of the proposed PD and the Au/Cr–GaN–Au/Cr MQW PD in different bias voltages under illumination with different wavelengths (405 nm, 450 nm, and 520 nm). **f** The fitting parameters *β* and the specific detectivity of the proposed PD and the Au/Cr-GaN-Au/Cr MQW PD under illumination with different wavelengths (405 nm, 450 nm, and 520 nm).

To understand the impact of interface quality, i.e. defect density or trap level, on device performance, Fig. 3 exhibits the noise characteristics of the MXene-GaN-MXene MQW PD and the Cr/Au–GaN–Cr/Au MQW PD at various applied bias voltages under different illumination conditions. As shown in Fig. 3a-d, all the measured noise spectral densities exhibit a linear decrease in the logarithmic coordinate axis, indicating that the 1/f noise dominates up to 100 kHz. Though origins of 1/f noise sources are not well understood up to now, it is believed that mobility fluctuation within the depletion region in forward bias voltage and Schottky barrier height fluctuations in reverse bias voltage are the major contributors^33^. The 1/f noise can be described by the Hooge empirical law^34^:

4 $$S_{{{\mathrm{I}}}} \propto \frac{{I^\beta }}{{f^\alpha }}$$

where $S_{{{\mathrm{I}}}}$ is the noise spectral density, $I$ the total device current, and $f$ the frequency. The exponents $\alpha$ approximately equals to 1 and $\beta$ varies from 1.5 to 2. As shown in Fig. 3a–d and Fig. S3a–b, the noise of the device using van der Waals junctions is lower than the Cr/Au counterpart for all cases, especially at low bias voltages. This is consistent with the Hooge empirical law that a large reverse current results in large 1/f noise. The photocurrent of the Cr/Au–GaN–Cr/Au MQW PD is negligible compared with its dark current so the corresponding noise spectral density is not changed much under illumination. However, as shown in Fig. 3a, b, the current under illumination of the MXene-GaN-MXene MQW PD is two orders of magnitude larger than in the dark but the noise spectral density only increase about one orders of magnitude, which may be attributed to a low level of interface imperfections and hence a smaller Schottky barrier height fluctuation. To quantitatively verify our hypothesis, the exponents $\alpha$ and $\beta$ are extracted into Fig. 3e, f, respectively. Parameter $\alpha$ varies between 0.98 and 1.02 under different conditions, exhibiting a standard 1/f noise characteristics of the two devices. Parameter $\beta$ for the device using the Cr/Au electrodes varies ~1.6 while for the device using MXene-GaN van der Waals junctions fluctuates more closer to 2. $\beta$ of Schottky barrier diodes under reverse bias is 2 and for surface trapping-limited 1/f noise, a typical value of $\beta$ is ~1 while for volume trapping-limited 1/*f* noise $\beta$ ≅ 1.5–2^35–37^. As mentioned afore, it is safe to conclude that the Cr/Au–GaN interface has a higher density of defects than the MXene-GaN interface, so the noise component corresponding to surface trapping is more, leading to a lower $\beta$ for the Cr/Au–GaN–Cr/Au MQW PD. However, in the MXene-GaN-MXene MQW PD, the Schottky barrier height fluctuations caused by the defect in the InGaN/GaN MQW is the major contributors, resulting in the corresponding $\beta$ close to 2. The fitting parameters agree well with the experiments and our hypothesis.

The specific detectivity, $D^ \ast$, is a figure of merit to evaluate the ability to detect week light signal. It is the inverse of the noise equivalent power ($NEP$) normalized by the absorption area and operation bandwidth, which can be expressed as:

5 $$D^ \ast = \frac{{\sqrt {A \cdot {\Delta}f} }}{{NEP}} = \frac{{R_{SNR = 1}\sqrt {A \cdot {\Delta}f} }}{{I_{{{{\mathrm{noise}}}}}}}$$

where $A$ is the absorption area and ${\Delta}f$ is the operation bandwidth. $R_{SNR \,=\, 1}$ is the responsivity when the signal-to-noise ratio (*SNR*) equals 1. This restriction is often forgotten in many literatures. Though it is hard to measure the $R_{SNR = 1}$, it can be calculated by the fitting parameters obtained afore. $I_{{{{\mathrm{noise}}}}}$ is the noise current which can be obtained by integrating the noise spectral density. A more accurate calculation method of the $D^ \ast$ is given in Part III of the supplementary information. As shown in Fig. 3f, the specific detectivities of MXene-GaN-MXene MQW PD reach 1.93 × 10^12^, 1.13 × 10^12^, and 1.76 × 10^10^ Jones for 405 nm, 450 nm, and 520 nm light, respectively. However, the specific detectivities of Cr/Au–GaN–Cr/Au MQW PD are only 1.40 × 10^8^, 5.78 × 10^7^, and 1.82 × 10^7^ Jones.

**Fig. 4:**
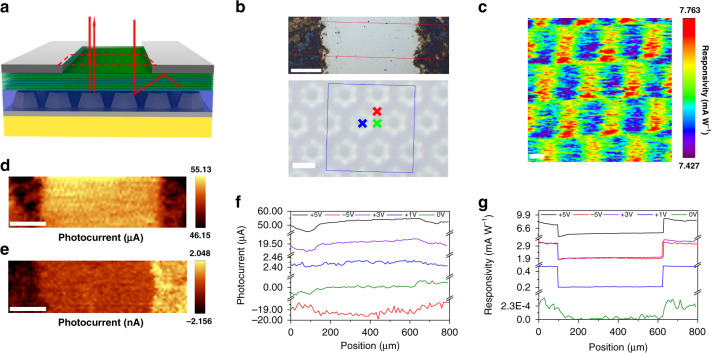
Photocurrent mapping. **a** Schematic diagram of normal incident light hitting on the proposed PD. **b** Low magnification (scale bar: 200 μm) and high magnification (scale bar: 3 μm) optical images of the proposed PD. Inside the red box is the large area photocurrent mapping region. Inside the blue box is the finer area responsivity mapping region. **c** The Responsivity mapping inside the blue box when the proposed PD at +5 V bias (scale bar: 1 μm). The large photocurrent mapping image inside the red box when the proposed PD at **d** +5 V and **e** 0 V biases (scale bar: 200 μm). **f** The photocurrent **g** and the responsivity distribution of the cut across from cathode to anode when the proposed PD at different bias (+5 V, −5V, +3 V, +1 V, and 0 V). The wavelength and optical power used in the mapping measurement are 532 nm and 10.42 mW, respectively.

To illustrate the promise of van der Waals junctions for efficient photocarrier collection, photoresponse mapping measurements were conducted on the MXene-GaN-MXene MQW PD as shown schematically in Fig. 4a. Figure 4b shows the low magnification and high magnification optical images of the device surface. The substrate patterns can be observed through the transparent nitride epilayers. Large area photocurrent mapping of the PD was performed at different biases, as shown in Figs. 4d, e and S4a–c. The line profiles of the photocurrent and responsivity distribution from the cathode to anode are extracted and plotted in Fig. 4f, g. Without any external bias, the photocurrent near the MXene electrodes is more dominant while the signal from the absorption region is marginal, as shown in Fig. 4e. In this case, only photon-generated carriers near the MXene electrodes can be collected by the external circuit and form a photocurrent with opposite signs due to the built-in electric field of the MXene-GaN Schottky junctions. When biased at different voltages, the MXene electrode region always show lower photocurrent signal compared with the absorption region. According to the reflection-transmission-absorption spectra of MXene shown in Fig. S2e, this is because the MXene electrodes absorb or reflect ~39% of the incident light. About 61% of the incident light passes through the MXene film and arrives the nitride layers. Additionally, Fig. 4f shows that no matter which side is reversely biased, the photocurrent in the absorption region always gradually increases from the anode side to the cathode side. As mentioned before, most of the electric potential drops at the cathode side so under the assistant of the large built-in electric field, the region near the cathode side can collect photoexcited carriers more efficiently. Though only part of the incident light can be absorbed by the MQWs under the MXene films, higher responsivity is achieved as shown in Fig. 4g in the MXene-GaN contact region, as a result of the vertical built-in electric field and the excellent weak light detecting ability of the proposed PD. Moreover, the photocurrent mappings in Figs. 4d, e and S4a–f make it evident that an asymmetric MSM diode is formed and explains the asymmetric IV curves shown in Fig. 2a.

Interestingly, the spatial photocurrent mapping of the MXene-GaN-MXene MQW PD shows localized photocurrent enhancement. Thanks to the formation of the high-quality MXene-GaN van der Waals junctions, it is possible to suppress the dark current and hence distinguish minor spatial variation of the photocurrent in the order of nanoamps. As depicted in Fig. 4c, the high-resolution responsivity mapping exhibits the same pattern as the sapphire substrate. The areas corresponding to the space between hexagonal nanopatterns show higher responsivity. This can be explained by the fact that normal incident light on the edge of hexagonal nanopatterns will be scattered in various directions. Some of photons with large incident angle experience total internal reflection at the GaN/air interface and are reflected back to the MQW region to excite carriers again, resulting in enhanced responsivity. The normal light hit right on the nanopatterns experiences less scattering, leading to inferior responsivity. Again, it is exactly the high-quality MXene-GaN van der Waals metal-semiconductor junction that leads to the fine distinction of photocurrents in different light absorption regions. As a result, the responsivity distribution can be observed and the light absorption enhancement can be confirmed.

**Fig. 5:**
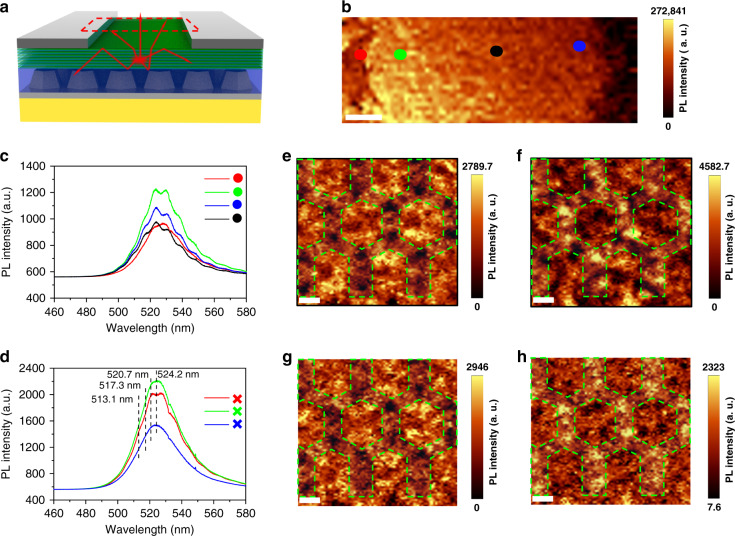
Photoluminescence characteristics. **a** Schematic diagram of photoluminescence in the photodetector. **b** Large area photoluminescence mapping image inside the red box (scale bar: 100 μm). **c** Photoluminescence spectra of four representative position marked by the black, green, red, and blue dot in Fig. 5b. **d** Photoluminescence spectra of three representative position marked by the green, red, and blue cross in Fig. 4b. The photoluminescence intensity mapping of the blue box region in Fig. 4b at the extracted Fabry–Pérot interference wavelengths of **e** 524.2 nm (trough), **f** 520.7 nm (peak), **g** 517.3 nm (trough), and **h** 513.1 nm (peak). The scale bar is 1 μm. The wavelength and optical power used in the mapping measurement are 532 nm and 10.42 mW, respectively.

To gain further insights into the localized photocurrent enhancement, micro-photoluminescence mapping is performed as illustrated in Fig. 5a. The photoluminescence intensity in the light absorption region gradually abated from the left side to the right side, implying an asymmetric spatial distribution of electric field near the two electrodes, in good agreement of the electrical measurements. Photoluminescence (PL) spectra of four representative positions, marked as black, green, red, and blue dots in Fig. 5b are shown in Fig. 5c. Interestingly, intensity undulations are observed in the PL spectra. We attribute this phenomenon to the Fabry–Pérot interference between the GaN/PSS interface and the GaN/air interface^38,39^. The refraction index of GaN is ~2.5 while the sapphire is ~1.8^40^. An intrinsic cavity is formed between the two interfaces, within which rays were multiply reflected to produce multiple transmitted rays. Therefore, intensity undulations appear in the PL spectra due to the Fabry–Pérot interference. A high-resolution photoluminescence mapping was performed. PL spectra measured from three representative positions in Fig. 4b: at the intersection of three nanopatterns (marked red), between two nanopatterns (marked green), and on a hexagonal nanopattern (marked blue), are plotted in Fig. 5d. The intensity undulations in the three PL spectra are less obvious than those extracted from the largescale photoluminescence mapping. As illustrated in Fig. 5a and reported in many literatures^41–43^, the patterned substrate can enhance the light extraction by scattering light in different directions. When the finer photoluminescence mapping is executed, the laser spot is highly focused (lateral resolution of ~300 nm) and in this case, light experienced less scattering. At the intersection of three nanopatterns, as a result of more severe scattering, the emission measured at the red cross shows more distinct Fabry–Pérot interference compared with the other two cases. For the same reason, a reduced PL intensity was measured on the hexagonal nanopattern (blue cross position). To confirm our hypothesis, PL mapping was performed at the extracted interference peaks of 520.7 nm and 513.1 nm and the interference troughs of 524.2 nm and 517.3 nm, as shown in Fig. 5d. The PL intensity integration width is 5 nm. The results are given in Fig. 5e–h and exhibit clear distribution of emission intensities following the sapphire nanopatterns. It verifies the Fabry–Pérot interference and enhancement of light extraction at the edge of the hexagonal nanopatterns. Therefore, the substrate nanopatterns also affect the photocurrent distribution and enhance the responsivity of the normal incident light, similar to the local enhancement in the light extraction. The localized light focusing and enhancement might be employed to construct large format focal plane arrays with submicron pixels for high-resolution imaging.

**Fig. 6:**
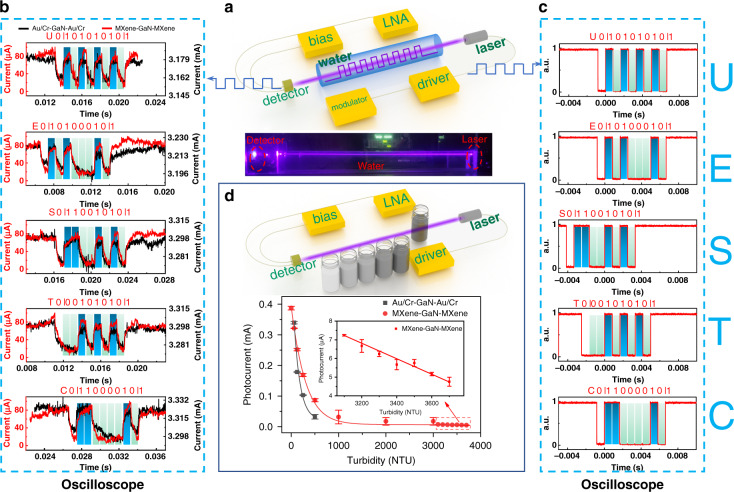
The underwater wireless optical communication system (UWOCS) and turbidity sensing system (TSS). **a** The schematic of an underwater wireless optical communication system. **b** Signals received by the proposed PD and the Cr/Au–GaN–Cr/Au MQW PD in the UWOCS. **c** Signal waveforms transmitted through the laser. **d** The photocurrent excited by the transmitted light in the TSS versus different turbidity. The inset is the schematic of TSS. The red and black lines are the fitted curves.

To verify the high performance of the MXene-GaN-MXene MQW PD device, it is also used to demonstrate underwater wireless optical communication and turbidity sensing. An underwater wireless optical communication system (UWOCS) is illustrated in Fig. 6a. The data is transmitted into a modulator to produce modulated electric signal. Then the electric signal was sent to a driver to drive the laser diode. The OOK modulated 405 nm light produced from the laser diode travels through the water and detected by the PDs. The photocurrent signal is amplified by a low noise amplifier (LNA). The UWOCS is set to work at 1200 baud. After passing through a 60-cm long water channel, the test signals received by the proposed PD and the Au/Cr-GaN-Au/Cr MQW PD can be directly recognized without any wave-shaping circuit, as shown in Fig. 6b and as expected, the proposed PD exhibits much lower dark current and higher photocurrent. Here, a character string, “UESTC”, is converted into binary data and modulates the laser for transmission, as shown in Fig. 6c. The normalized spectra of the received signals were calculated and given in Fig. S5a–e. In all spectra, the components ranging from DC to 20 kHz of the two PDs are about the same magnitudes while ranging from 20 to 50 kHz, the component magnitudes of the proposed PD are lower than its counterpart, which implies the signals received by the proposed PD have lower noise. Additionally, a turbidity sensing system (TSS) schematic is shown in the inset of Fig. 6d. The architecture of the TSS is similar to the UWOCS but the modulator is removed. While a 405-nm light beam travels through the standard turbidity solution in different concentrations, various degrees of light scattering are presented in different standard solutions. As a result, the amount of the light arriving the PD is different. As shown in Fig. 6d, the proposed MXene-GaN-MXene MQW PD shows a large detection range from 0 to 4000 NTU and it offers a high detection resolution of ~100 NTU in high turbidity, even though only a single channel measuring transmitted light is employed. However, the Au/Cr-GaN-Au/Cr MQW PD cannot distinguish the scattered light when the turbidity is or higher than 1000 NTU due to its lower detectivity, as shown in Figs. 6d and S6.

## Discussion

We have successfully fabricated and demonstrated a MXene-GaN-MXene MQW PD with enhanced responsivity and reduced dark current. Thanks to the facile synthesis and functionalization of Ti_3_C_2_T_x_ films, high quality electrodes can be prepared on a wide range of semiconductors with low cost. Particularly, the van der Waals MXene-GaN interfaces obtained in this work suppress dark current and enhance the collection of photon-excited carriers due to the low density of chemical disorders and defects at the interfaces. The low dark current of the MXene-GaN-MXene MQW PD, three orders of magnitude lower than that of the Cr/Au–GaN–Cr/Au device, makes it evident that high quality van der Waals junctions are formed. Compared to the traditional Cr/Au–GaN–Cr/Au MQW PD, the 1/*f* noise spectral intensity of the MXene-GaN-MXene MQW PD is reduced and the corresponding fitting parameters verify the Cr/Au–GaN interface has a higher density of traps than the MXene-GaN interface. The measured responsivity and specific detectivity reach as high as 64.6 A/W and 1.93 × 10^12^ Jones, respectively, under *λ* = 405 nm illumination. The proposed PD showed high responsivity and low noise in the blue-green light spectrum range, which can be potentially used in underwater optical communication and detection. Moreover, it is found that the substrate nanopatterns can locally enhance the responsivity by scattering the normal incident light into different directions. The high-quality MXene/GaN interfaces enable identification of photocurrent distribution with fine distinction of nanoamps in submicron scale. The mechanism of local photocurrent enhancement is supported by micro-PL mapping. An underwater optical wireless communication system and a turbidity sensing system equipped with the MQW PD are also demonstrated. This work exhibits good potential of van der Waals metal-semiconductor junctions for high performance photodetection by combining the advantages of 2D MXene materials and the conventional III–V materials.

## Materials and methods

### Synthesis of Ti_3_C_2_T_x_ MXene solution

LiF (0.67 g) was slowly dissolved in HCl (6 M, 10 mL) solution with continuous stirring for 5 min. Subsequently, Ti_3_AlC_2_ MAX powder (1 g) was gradually added into the etchant under gentle stirring, followed by magnetic stirring for 24 h at the room temperature. Next, the acidic mixture was transferred to a centrifuge tube and centrifuged at 3500 rpm. The obtained suspension was repeatedly washed with deionized (DI) water and centrifuged until reaching a neutral pH. The Ti_3_C_2_T_x_ sediment was collected to redisperse in DI water and sonicated for 3 h under the Argon atmosphere. At last, the solution was centrifuged at 3500 rpm for 1 h and the supernatant solution was collected for further experiment.

### Growth of the InGaN/GaN multiple quantum well

A (0001) patterned sapphire wafer is employed as the growth substrate, on which hexagonal nanopatterns are fabricated by the standard lithography process and wet-etching. The InGaN/GaN MQW was grown heteroepitaxially by atmospheric pressure metal organic chemical vapor deposition (MOCVD) on the patterned sapphire substrate (PSS). The n-side of the device structure consisted of a 2.4 μm unintentionally doped (UID) GaN template layer, and a 1.5 μm Si-doped n-GaN layer ([Si] = 4 × 10^18^ cm^−3^). The active region consisted of an Si-doped eight period MQW that was grown in two steps: the first step consisted of the growth of a 3-nm InGaN QW with ~25% indium content. The second step consisted of ramping up the temperature 100 °C higher than the QWs in 60 s, and the growth of a 10-nm high temperature (HT) GaN barrier.

### Fabrication of the MXene-GaN-MXene structure

The epitaxial wafer was washed by DI water. While the wafer is still wet, a polyvinyl chloride (PVC) electrostatic film with electrode patterns was sticked on it. After the DI water dried up, the as-prepared Ti_3_C_2_T_x_ MXene solution was dropped on the mask to cover the carved patterns and then let it dry naturally. The PVC electrostatic film was removed after the formation of Ti_3_C_2_T_x_ MXene film. At the same time, a Cr/Au–GaN–Cr/Au MSM photodetector was fabricated by using the same mask. In this case, 5 nm Cr adhesion layer and a 50-nm Au contact layer were deposited on the surface of the MQW epilayers successively through electron-beam evaporation. Finally, all the PDs were annealed at 300 °C in the argon atmosphere.

### Device characterization

The photoresponse characteristics of the device were measured by a semiconductor parameter analyzer (4200A-SCS, Keithley) and a probe station (LT3, Advanced Research Systems). The noise spectral density was measured by a semiconductor parameter analyzer with a noise testing module (FS-Pro, Primarius). Different TTL modulated laser diodes with wavelengths of 405 nm, 450 nm, and 520 nm were used as the light sources for photoresponse measurements. The linear dynamic range (${\mathrm{LDR}}$) of photodetectors defined as:

6 $${\mathrm{LDR}} = 10 \times {\mathrm{log}}_{10}\frac{{L_{{{{\mathrm{max}}}}}}}{{L_{{{{\mathrm{min}}}}}}}[dB]$$

where $L_{{\mathrm{max}}}(L_{{\mathrm{min}}})$ is the maximum (minimum) incident optical power when the photocurrent increases linearly with the optical power. Function signal generator (AFG1062, Tektronics) was used to generate the modulating signal. All the optical power were determined by an irradiatometer (FZ-A, Shenzhen Chuangxin Instrument Inc.). Photoluminescence and photocurrent mapping was executed through a confocal laser scanning microscope (Alpha300 RA, WITec).

## Supplementary information

Supplementary Information

## Data Availability

The data that support the findings of this study are available from the corresponding author upon reasonable request.
