# Supplementary material for: MXene-GaN van der Waals metal-semiconductor junctions for high performance multiple quantum well photodetectors
Source: Light Sci Appl. 2021 Sep 2;10:177. doi: 10.1038/s41377-021-00619-1 (PMC8410839; doi:10.1038/s41377-021-00619-1)
Supplement: Supplementary file 1 — Supplementary Information [file 41377_2021_619_MOESM1_ESM.docx]

Supplementary Information for: MXene-GaN van der Waals Metal-Semiconductor Junctions for High Performance Multiple Quantum Well Photodetectors

Lingzhi Luo^1^, Yixuan Huang^1^, Keming Cheng^1^, Abdullah Alhassan^2^, Mahdi Alqahtani^3^, Libin Tang^4^, Zhiming Wang^1^, Jiang Wu^1, 5,*^

1 Institute of Fundamental and Frontier Sciences, University of Electronic Science and Technology of China, Chengdu 610054, China

2 Materials Department, University of California, Santa Barbara, California 93106, United States

3 King Abdulaziz City for Science and Technology (KACST)

4 Kunming Institute of Physics, Kunming 650223, Yunnan, China

5 State Key Laboratory of Electronic Thin Films and Integrated Devices, University of Electronic Science and Technology of China, Chengdu, Sichuan 610054, China

* Email: [jiangwu@uestc.edu.cn](mailto:jiangwu@uestc.edu.cn)

**Part I: Leakage mechanism in GaN Schottky interfaces**

**Part Ⅱ: Extended experimental data of sample characterization**

Fig S1: Extended *I-V* curves

Fig S2: Extended photoresponse characterization

Fig S3: Extended noise characterization

Fig S4: Extended photocurrent mapping

Fig S5: Normalized spectra of the electric signals received by detectors

Fig S6: Response of the Au/Cr-GaN-Au/Cr MQW PD in the turbidity sensing system

**Part** **ⅡI: Calculation of specific detectivity (**$\boldsymbol{D}^{\mathbf{*}}$**)**

TABLE S1: PARAMETERS FOR THE CALCULATION OF $D^{*}$

**Reference**

Part I: Leakage mechanism in GaN Schottky interfaces

The reverse biased dark current considering surface defects can be expressed as^1^:

| $I_{\mathrm{dark}}=A\cdot\frac{4\pi qm^{*}}{h^{3}}\int_{0}^{\infty} T\left( E_{x} \right) \int_{0}^{\infty} \left[ f_{s}\left( E_{x}+E_{p} \right)-f_{m}\left( E_{x}+E_{p} \right) \right]dE_{p}dE_{x} \left[ Amp \right] (1)$ |
| --- |

where $m^{*}$ is the effective mass, $h$ is the Plank constant, $T\left( E_{x} \right)$ is the tunneling probability, $E_{x}$ and $E_{p}$ are the energy components normal and parallel to the Schottky barrier, respectively, and $f_{m}(E)$ and $f_{s}(E)$ are the Fermi-Dirac distribution functions for metal and semiconductor, respectively. $T\left( E_{x} \right)$ can be calculated using Wentzel–Kramers–Brillouin approximation:

|  | $T(x)=exp[-2\frac{\sqrt{2m^{*}}}{\hbar} \int_{x_{1}}^{x_{2}} \sqrt{\phi\left( x \right)-E_{x}}dx$] | $(2)$ |
| --- | --- | --- |

where $\phi\left( x \right)$ is the potential distribution and $x_{1}$ and $x_{2}$ are classical turning points. According to Ref. 1, deep defect donors with an exponentially decaying spatial distribution near the interface thin the Schottky barrier described in $\phi\left( x \right)$, and thus increase the dark current through thermionic field emission.

Part Ⅱ: Extended experimental data of sample characterization


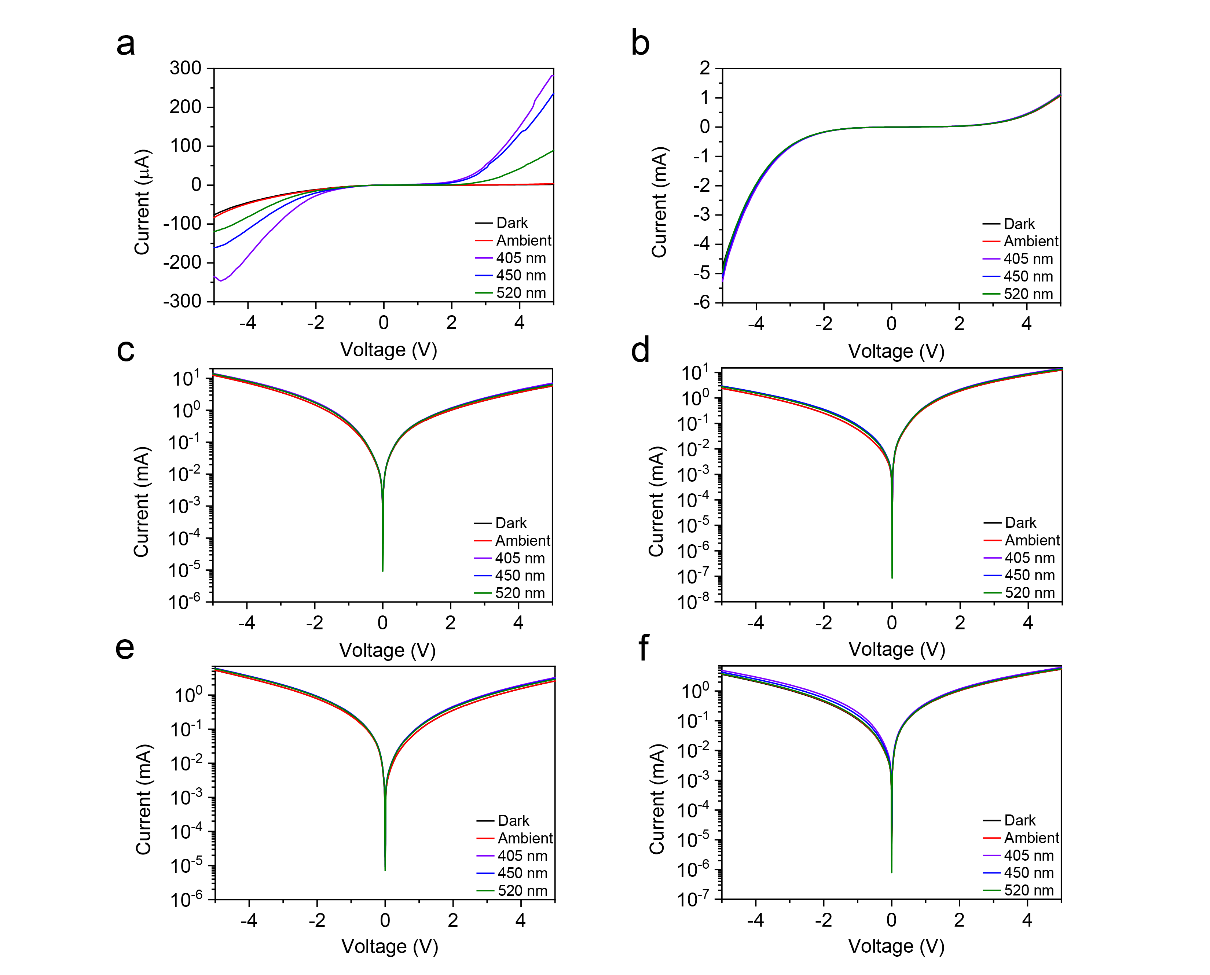


**Fig S1: Extended I-V curves.** *I*–*V* curves of **a** the proposed PD and **b** the Au/Cr-GaN-Au/Cr MQW PD measured in the dark, ambient and under illumination with different wavelengths (405 nm, 450 nm, 520 nm). **c-f** *I*–*V* curves in the logarithmic coordinate axis of another 4 Au/Cr-GaN-Au/Cr MQW PDs.


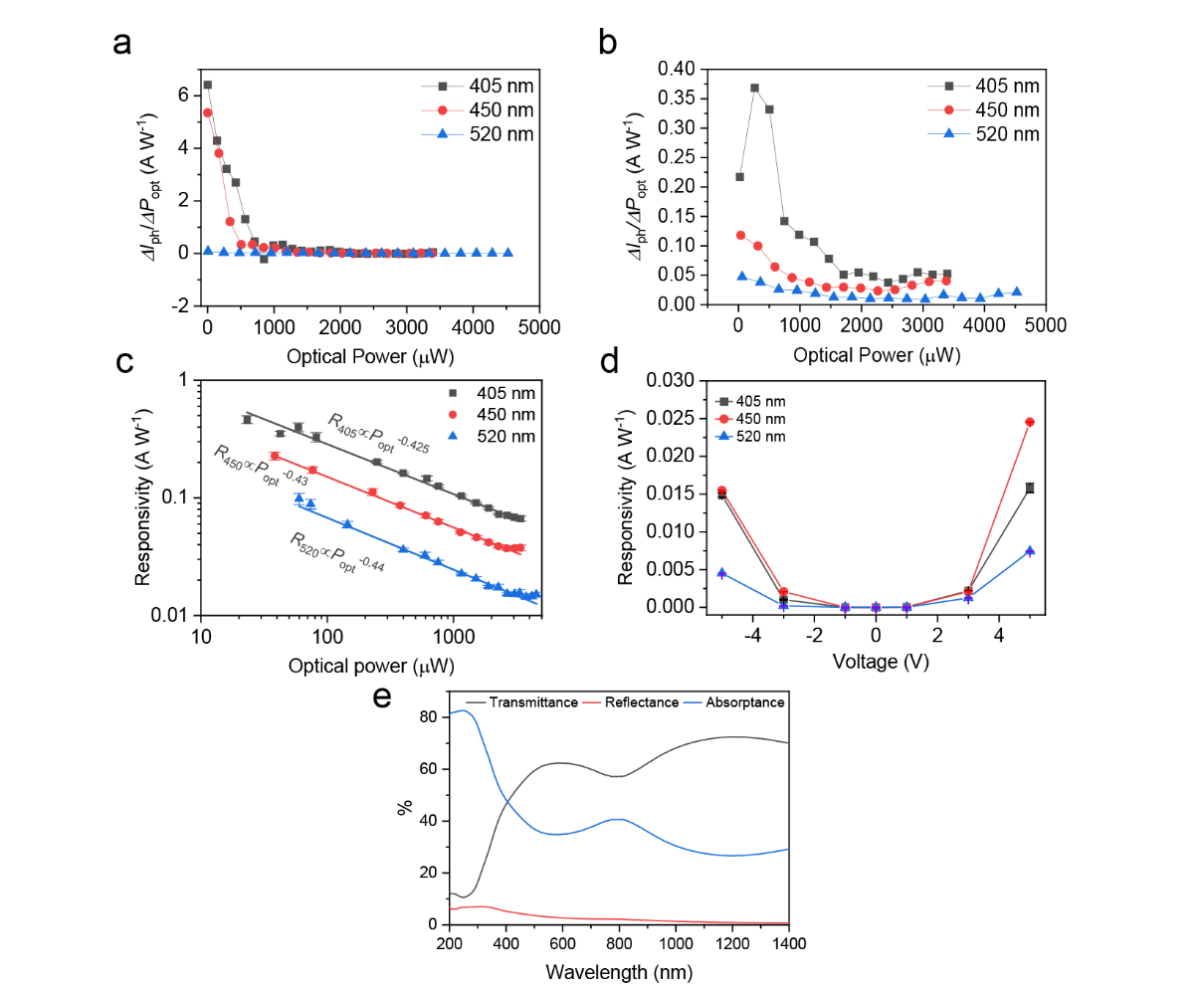


**Fig S2: Extended photoresponse characterization.** $\Delta I_{\mathrm{ph}}/\Delta P_{\mathrm{opt}}$ versus the incident optical power of **a** the proposed PD and **b** the Cr/Au–GaN–Cr/Au MQW PD under different illumination (405 nm, 450 nm, 520 nm). **c** The responsivity of the Cr/Au–GaN–Cr/Au MQW PD versus the incident optical power in the logarithmic coordinate. The fitted line and parameters are shown and marked in the figure. **d** The responsivity of the proposed PD at different biases under different illumination (405 nm, 450 nm, 520 nm). **e** The reflection-transmission-absorption spectra of the MXene film.

**
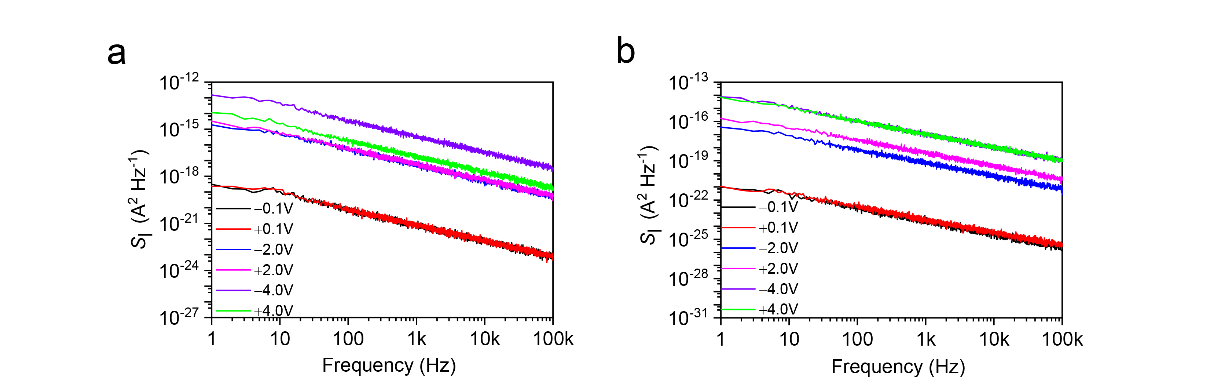
**

**Fig S3: Extended noise characterization.** Noise spectral density of **a** the Cr/Au–GaN–Cr/Au MQW PD and **b** the proposed PD at different biases and under illumination of a 450 nm laser diode.


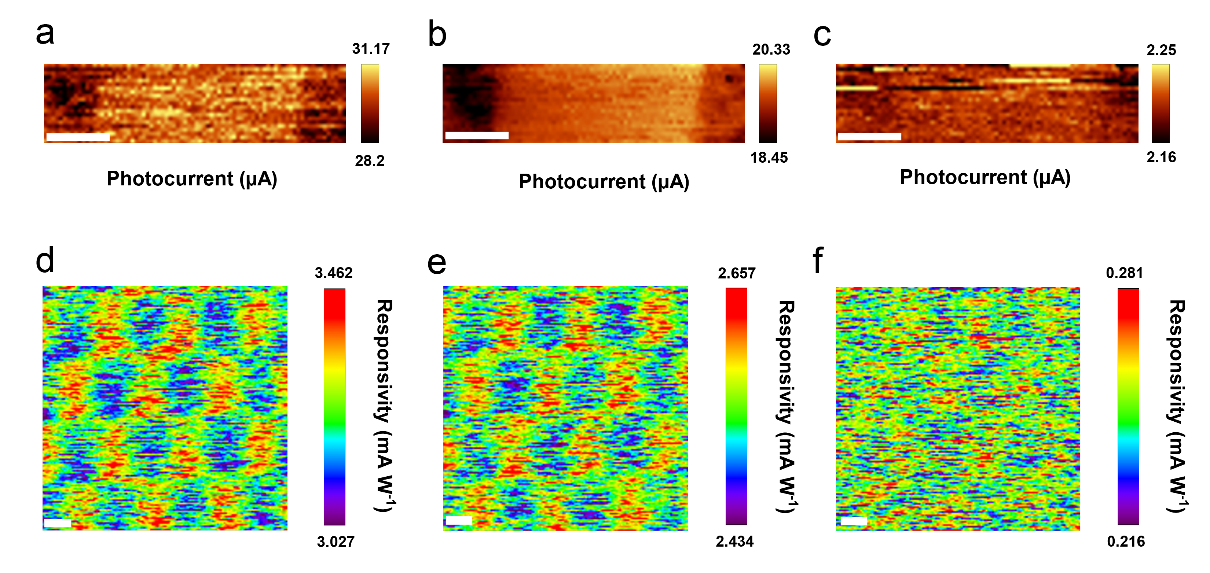


**Fig S4: Extended photocurrent mapping.** Photocurrent mapping images inside the red box when the proposed PD biased at **a** -5V **b** +3V and **c** +1V (scale bar: 200 μm). The high resolution responsivity mapping inside the blue box when the proposed PD biased at **d** -5V **e** +3V and **f** +1V (scale bar: 1 μm). The wavelength of the laser is 532 nm.


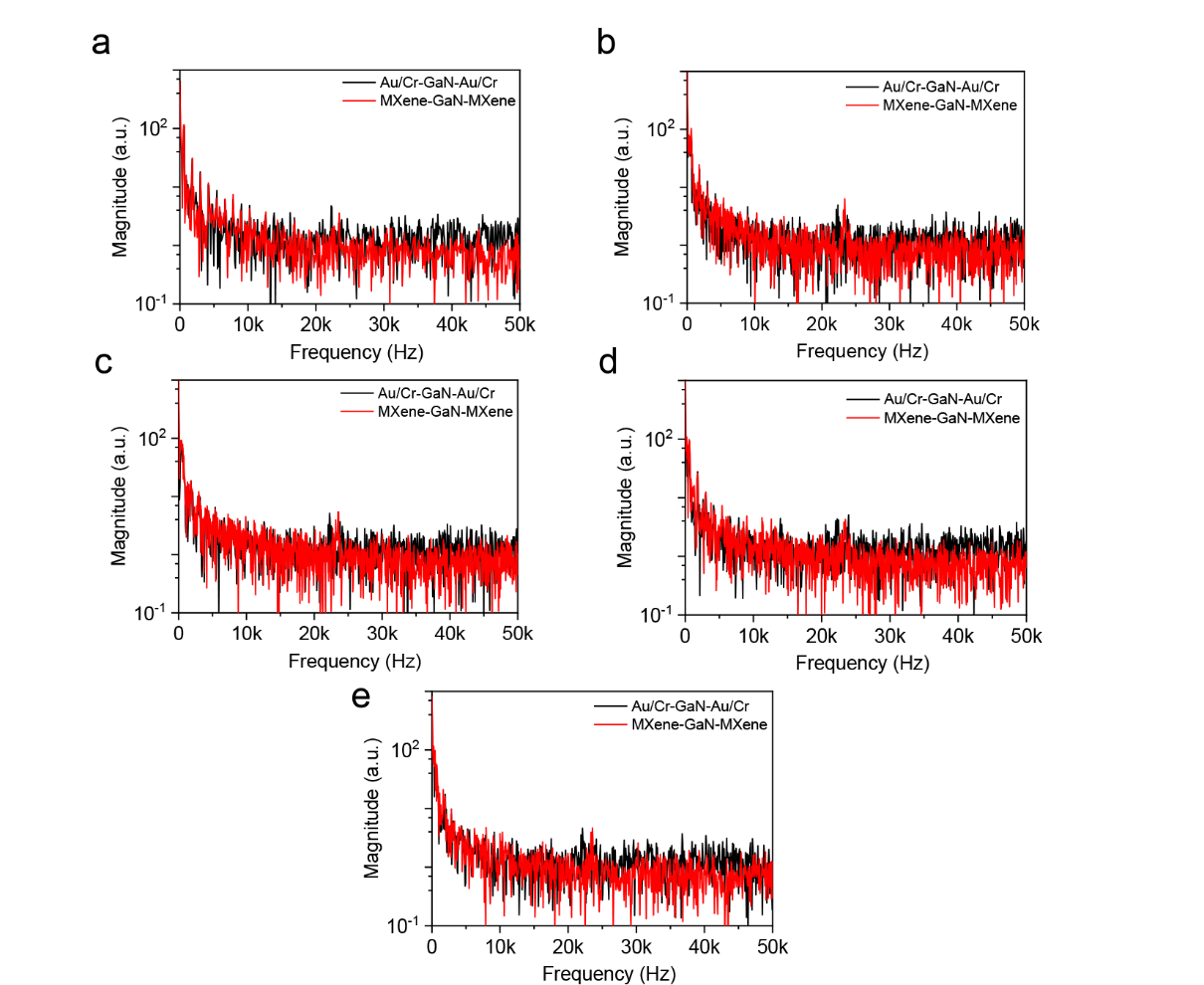


**Fig S5: Normalized spectra of the electric signals received by detectors .** The normalized spectra of the digital signals of character **a** U, **b** E, **c** S, **d** T and **e** C received by the proposed photodetector and the Au/Cr-GaN-Au/Cr MQW photodetector, respectively.


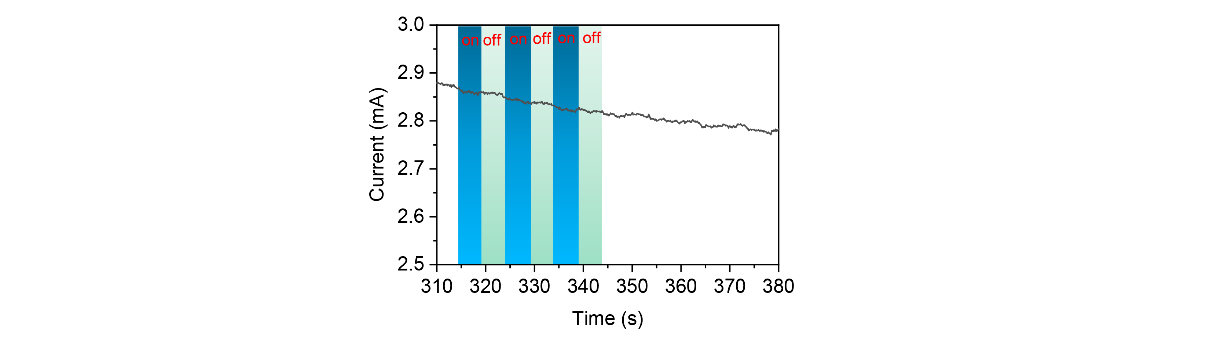


**Fig S6: Response of the Au/Cr-GaN-Au/Cr MQW PD in the turbidity sensing system.** The current response of the Au/Cr-GaN-Au/Cr MQW PD in the turbidity sensing system when the turbidity is 1000 NTU and the laser was turned on and off.

**Part ⅡI: Calculation of specific detectivity (**$\boldsymbol{D}^{\mathbf{*}}$**)**

When $SNR=1$, we have:

|  | $I_{\mathrm{noise}}=I_{\mathrm{ph}}$ | (3) |
| --- | --- | --- |

$I_{\mathrm{noise}}$ can be obtained by integrating the noise spectral density:

|  | $I_{\mathrm{noise}}=\sqrt{\int_{1}^{B} S_{I}ⅆf}=\sqrt{\int_{1}^{B} k\frac{I^{\beta}}{f}ⅆf}$ | (4) |
| --- | --- | --- |

where $I=I_{\mathrm{ph}}+I_{\mathrm{dark}}$ and $B$ is the operation bandwidth. $k$ and $\beta$ are the fitting parameters obtained from our experiments.

The responsivity can be expressed as:

|  | $R={10}^{\theta}\cdot P_{\mathrm{opt}}^{\gamma}=\frac{I_{\mathrm{ph}}}{P_{\mathrm{opt}}}$ | (5) |
| --- | --- | --- |

where $\theta$ and $\gamma$ are the fitting parameters obtained from our experiments.

By solving simultaneous equation (8)-(11), we can obtain $R_{SNR=1}$, then the specific detectivity can be calculated:

|  | $D^{*}=\frac{R_{SNR=1}\sqrt{A\cdot\Delta f}}{I_{noise}}$ | (6) |
| --- | --- | --- |

The parameters obtained from our experiments are shown in TABLE S1.

TABLE S1

PARAMETERS FOR THE CALCULATION OF $D^{*}$

|  | Au/Cr-GaN-Au/Cr MQW PD | | | MXene-GaN-MXene MQW PD | | |
| --- | --- | --- | --- | --- | --- | --- |
|  | 405 nm | 450 nm | 520 nm | 405 nm | 450 nm | 520 nm |
| *B* (Hz) | 1167 | 1167 | 1167 | 1167 | 1167 | 1167 |
| *A* (mm^2^) | 2.84 | 2.84 | 2.84 | 2.84 | 2.84 | 2.84 |
| *γ* | -0.425 | -0.43 | -0.44 | -0.61 | -0.62 | -0.56 |
| *θ* | -1.81874 | -2.11302 | -2.49147 | -2.732 | -2.929 | -3.091 |
| *β* | 1.51 | 1.632 | 1.571 | 1.679 | 1.774 | 1.676 |
| $k$ | ${10}^{-9.77}$ | ${10}^{-9.572}$ | ${10}^{-9.758}$ | ${10}^{-6.985}$ | ${10}^{-6.512}$ | ${10}^{-7.14}$ |

Reference

1. Hashizume, T., Kotani, J. & Hasegawa, H. Leakage mechanism in GaN and AlGaN Schottky interfaces. *Appl. Phys. Lett.* **84,** 4884–4886 (2004).
